# Supplementary material for: Urban scaling in Europe
Source: J R Soc Interface. 2016 Mar;13(116):20160005. doi: 10.1098/rsif.2016.0005 (PMC4843676; doi:10.1098/rsif.2016.0005)
Supplement: Electronic Supplementary Material [file rsif20160005supp1.pdf]

Electronic Supplementary Material

-

Urban Scaling in Europe

Luís M. A. Bettencourt[1,*], José Lobo[2]

[1]Santa Fe Institute, 1399 Hyde Park Rd, Santa Fe NM 87501, USA,

[2]School of Sustainability, Arizona State University, 800 Cady Mall, Tempe, AZ 85281, USA.

[*]To whom correspondence should be addressed: bettencourt@santafe.edu

I. Metropolitan Areas: Scaling Relations for Additional Variables

In this section we demonstrate the scaling properties of additional variables, reported in the main text.

Figure ?? show the behavior of Labor Productivity, the fraction of GDP attributable to employment. Points and lines follow the definitions of the main text. The data agree very well with the predictions from urban scaling theory for 102 European Metropolitan Areas. Fitting parameters are given in the captions. Similar scaling analyses and fitting results are also shown in Fig. ??, for Unemployment, and in Fig. ??, for CO_2 emissions of the same Metropolitan Areas.

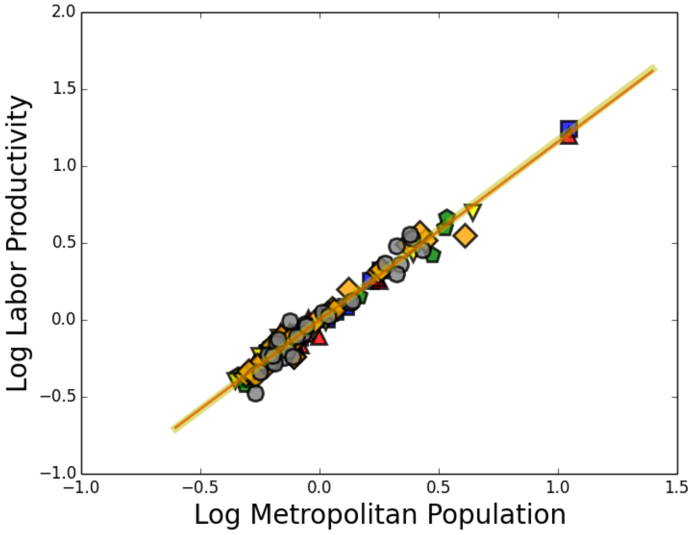

FIG. S1. Labor Productivity European Metropolitan areas (102 cities). The data has been centered in each nation. The gradient estimated via best fit (red line) corresponds to a scaling exponent $\beta = 1.16$ (95% Confidence Interval [1.12,1.20], $R^2 = 0.94$) statistically indistinguishable from the simplest prediction from urban scaling theory, for $\beta = 7/6$ (yellow thick line).

II. Structure of Residuals from Scaling

In this section, we present additional information for ranked lists of residuals of scaling best-fits. In order to present a summary analysis we focused on Europe-wide fits, obtained after centering the data in each nation as shown in the Figures of the previous section.

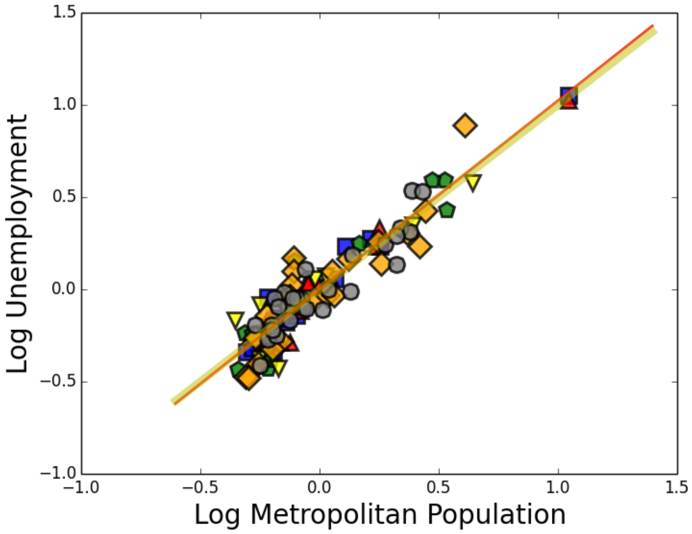

FIG. S2. Unemployment in European Metropolitan areas (102 cities). Note that unemployment measures something different from number of employed versus population as unemployment registers people wanting to find work that have not succeeded. A city can have more or fewer employed persons per capita without such number necessarily translating into unemployment. Similarly to employment, the best fit (red line) reveals an approximately linear slope (yellow line), with $\beta = 1.02$ (95% Confidence Interval [0.95,1.1], $R^2 = 0.77$). Berlin (rightmost orange diamond) shows high levels of unemployment in agreement with the comments made in the main text. See also residuals analysis in the next section.

Residuals of these fits are city size independent by construction. After centering they area also independent of their national contexts. For these reasons, these residuals can be used as *Scale-Invariant Metropolitan Indicators* ("SAMIs"), which allow a direct comparison between the performance of cities of different sizes.

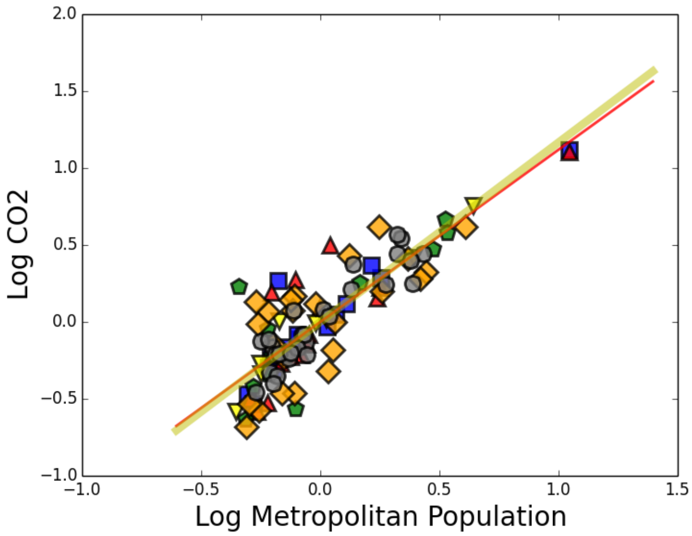

FIG. S3. Total CO_2 emissions of European Metropolitan areas (102 cities). The fit is not particularly clean and deviations in each place matter and must be better understood, see also residuals below. The best fit line (red) has slope $\beta = 1.12$ (95% Confidence Interval [0.98,1.26], $R^2 = 0.53$), a little below the simplest prediction from urban scaling theory with $\beta = 7/6$ (yellow), which assumes no transportation mode change or large building effects in energy savings. Perhaps because of such effects, the largest cities in Europe (Paris, London) fall slightly below both lines.

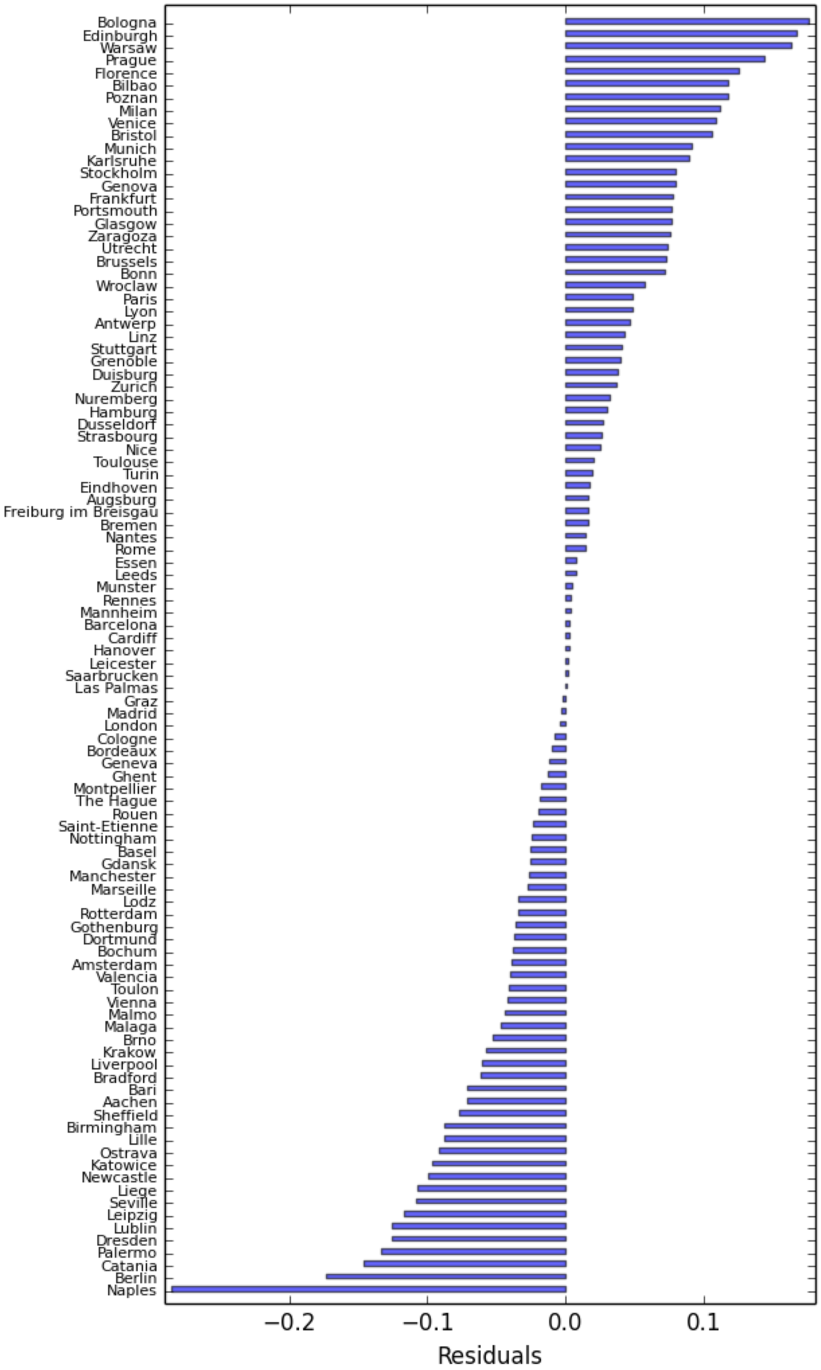

FIG. S4. Residuals for Metropolitan GDP, see main text (102 units). This ranked ordering confirms the statements made in the main paper that Naples, and other cities of Southern Italy (Catania, Palermo) are some of the most economically underperforming metropolitan areas in Europe, as are Berlin and other Metropolitan Areas in Eastern Germany (Dresden, Leipzig). On the upside, cities such as Bologna, Edinburgh, Prague or Bilbao show some of the most impressive economic performance, once their size and national context has been accounted for.

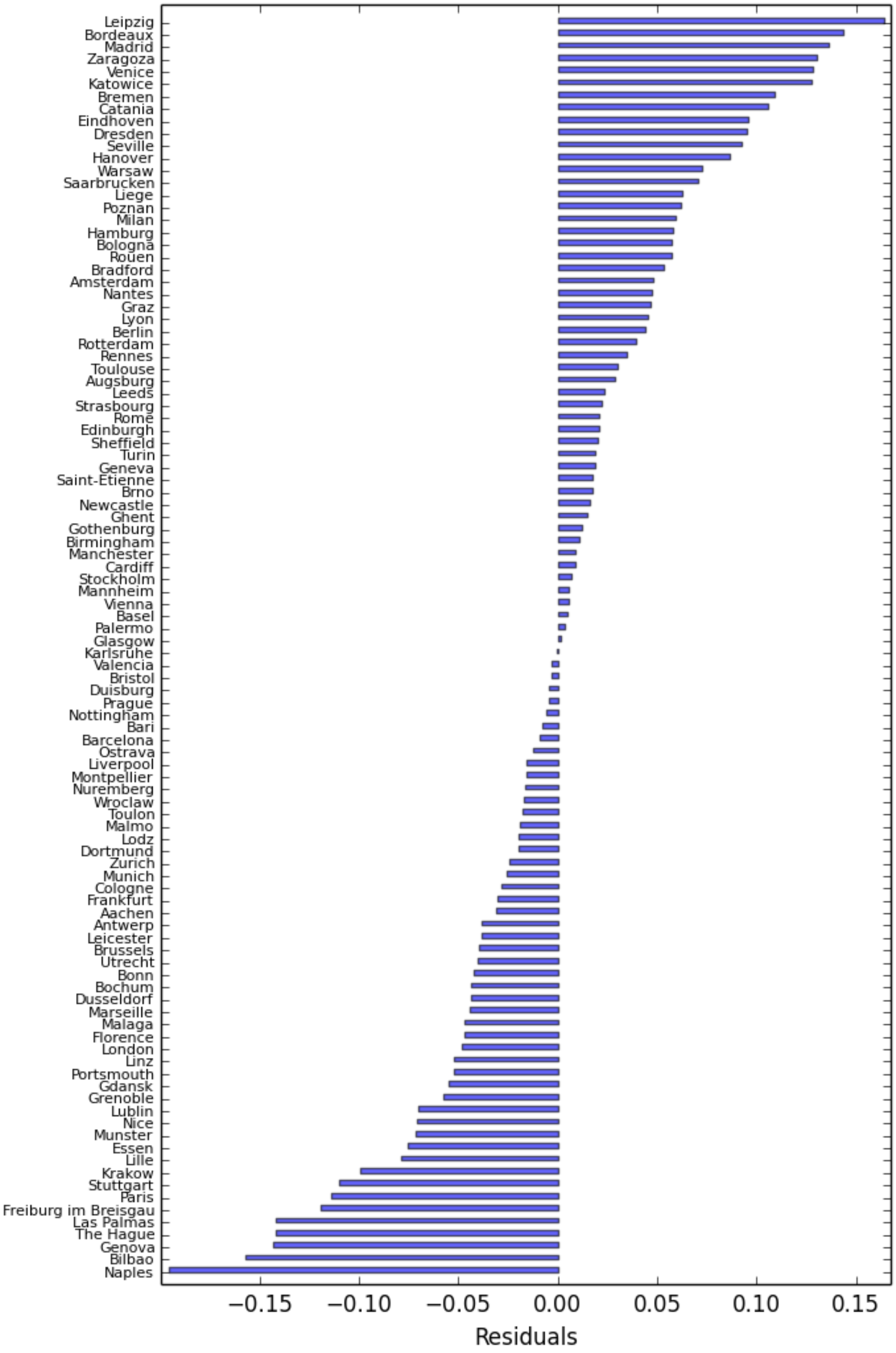

FIG. S5. Residuals for Urbanized Area, see main text (102 units). This confirms general statements made in the main text, about cities like Leipzig, Dresden and Madrid manifesting a very large urbanized area for their population size, while others, such as Naples, Bilbao, or Las Palmas, seem small.

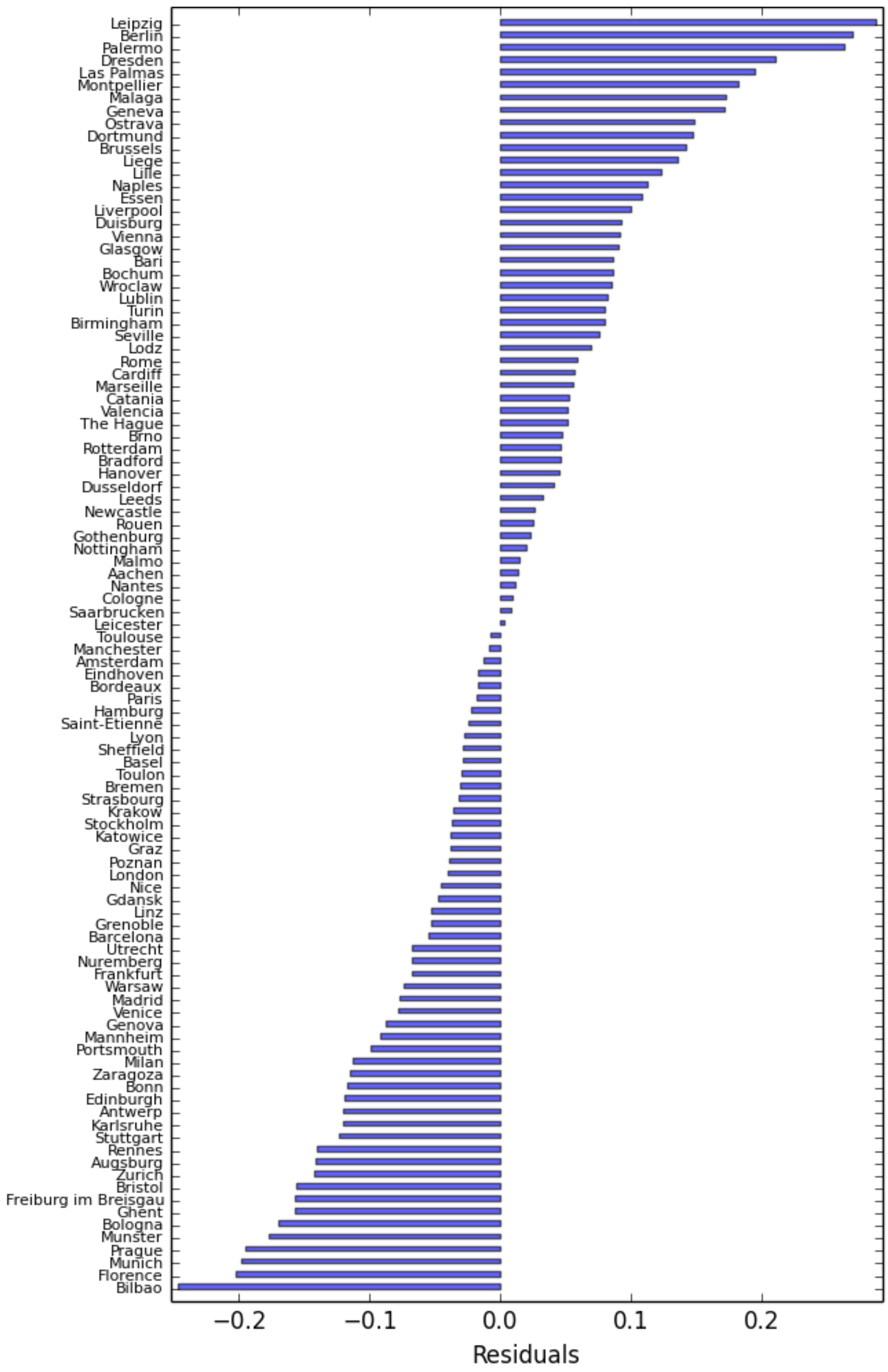

FIG. S6. Residuals for Unemployment in European Metropolitan Areas (102 units), see section above. This analysis confirms Berlin, Leipzig and Dresden again as exceptionally underperforming along this measure, not only within Germany but across Europe. Likewise for Montpellier and Lille, in France, and some cities of Southern Spain, such as Las Palmas and Malaga.

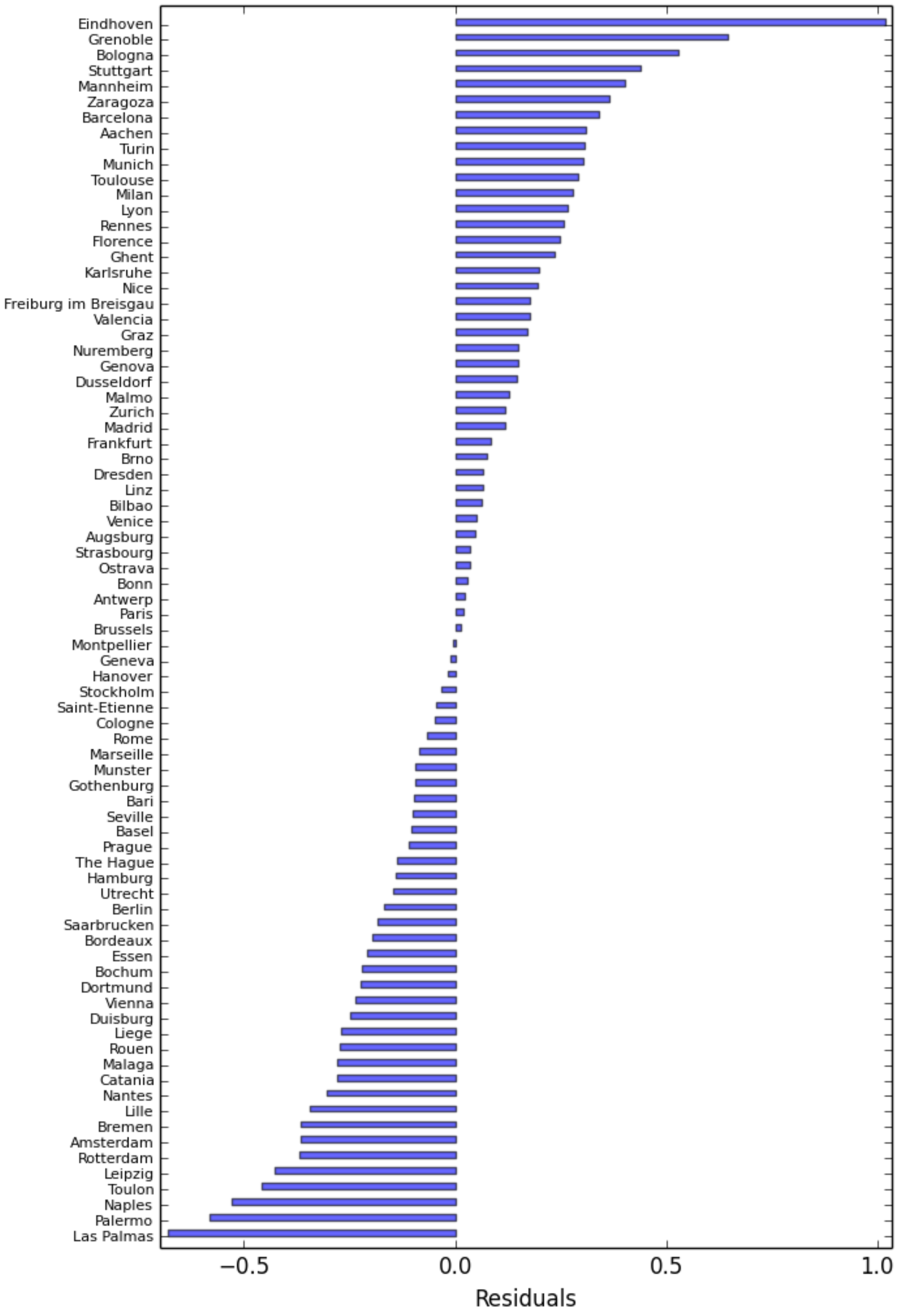

FIG. S7. Residuals for Patents (79 cities, excludes the UK and Poland). The ranking of Europe-wide residuals confirms several statements in the main text including the underperformance of Las Palmas, Palermo, Naples, Toulon and Leipzig. The list of the most inventive Metropolitan Areas in Europe is also curious: It is clearly headed by Eindhoven, followed by Grenoble, Bologna, Stuttgart, Mannheim, and Zaragoza, showing a healthy diversity of cities throughout Europe.

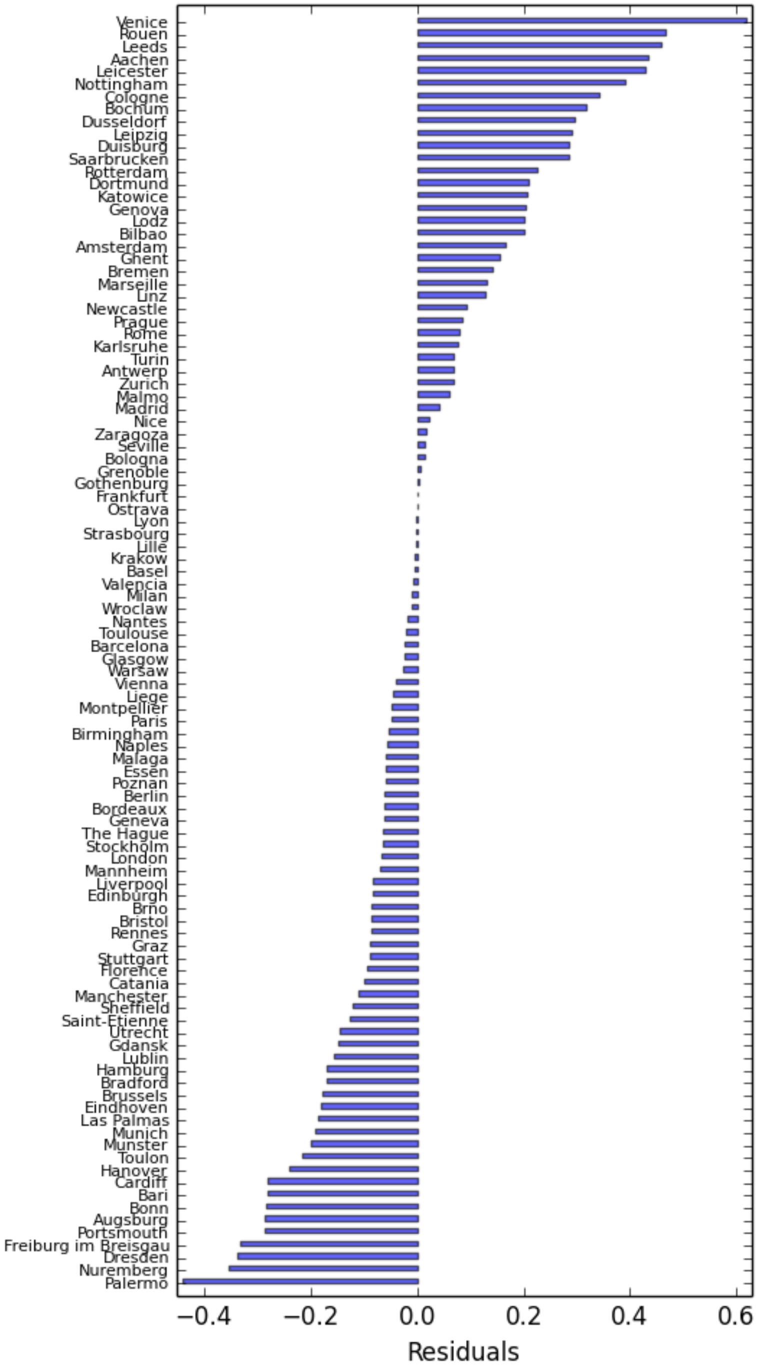

FIG. S8. Residuals for CO_2 emissions in European Metropolitan Areas (102 units). Note the large dispersion (magnitude of residuals is larger than for other quantities) across metropolitan areas, including many within the same nations, especially Germany, the UK and Italy. This suggests that energy systems and solutions towards decarbonization have a more local component than other urban variables. For example, German cities close to the Ruhr valley show some of the largest CO_2 emissions in Europe whereas others some of the lowest.
